# The Art of Designing Remote IoT Devices—Technologies and Strategies for a Long Battery Life

**DOI:** 10.3390/s21030913

**Published:** 2021-01-29

**Authors:** Gilles Callebaut, Guus Leenders, Jarne Van Mulders, Geoffrey Ottoy, Lieven De Strycker, Liesbet Van der Perre

**Affiliations:** ESAT-DRAMCO, Ghent Technology Campus, KU Leuven, 9000 Ghent, Belgium; gilles.callebaut@kuleuven.be (G.C.); guus.leenders@kuleuven.be (G.L.); jarne.vanmulders@kuleuven.be (J.V.M.); geoffrey.ottoy@kuleuven.be (G.O.); lieven.destrycker@kuleuven.be (L.D.S.)

**Keywords:** embedded design, energy management, energy-saving strategies, internet of things, low-power wide-area networks, low-power design, sensors

## Abstract

Long-range wireless connectivity technologies for sensors and actuators open the door for a variety of new Internet of Things (IoT) applications. These technologies can be deployed to establish new monitoring capabilities and enhance efficiency of services in a rich diversity of domains. Low energy consumption is essential to enable battery-powered IoT nodes with a long autonomy. This paper explains the challenges posed by combining low-power and long-range connectivity. An energy breakdown demonstrates the dominance of transmit and sleep energy. The principles for achieving both low-power and wide-area are outlined, and the landscape of available networking technologies that are suited to connect remote IoT nodes is sketched. The typical anatomy of such a node is presented, and the subsystems are zoomed into. The art of designing remote IoT devices requires an application-oriented approach, where a meticulous design and smart operation are essential to grant a long battery life. In particular we demonstrate the importance of strategies such as “think before you talk” and “race to sleep”. As maintenance of IoT nodes is often cumbersome due to being deployed at hard to reach places, extending the battery life of these devices is critical. Moreover, the environmental impact of batteries further demonstrates the need for a longer battery life in order to reduce the number of batteries used.

## 1. Introducing Opportunities and Challenges in the Design of Remote IoT Devices

The art of designing remote Internet of Things (IoT) devices with a long battery life requires, in the first place, a good understanding of the specific application, and a solid analysis of its requirements and deployment conditions. In this section, we provide illustrative examples that demonstrate the game-changing nature of remote IoT solutions for increasing efficiency in professional domains, to address societal problems, not in the least relating to our environment, as well as for leisure and cultural purposes. These use cases motivate the need for adequate technologies, and reveal their desired characteristics. Furthermore, we explain the challenges in designing remote IoT nodes, and introduce the concept, contributions, and structure of this paper.

### 1.1. Opportunities for a Diversity of Applications

Sensors observe physical phenomena and translate their measurements to electrical signals. When connected through long-range wireless interfaces, they can provide direct access to these measurements in remote environments and conditions. Such functionality can avoid the need for on-site checks, provide early and fast information on alarming situations, or simply give insights which was previously infeasible.

**Figure 1:**
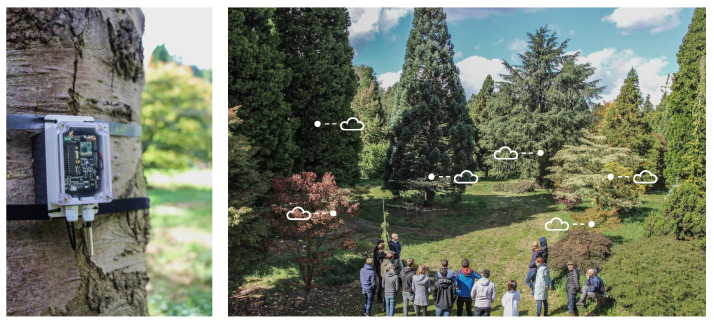
The ‘IoTree’ node [1] is designed and deployed for remote monitoring of a tree’s health.

Environmental monitoring is a domain where large-scale remote sensing can help to gain a better understanding in order to take more adequate measures to combat pollution and climate change. For example, precision farming based on the analysis of the actual conditions in the field can optimize the usage of fertilizers and agrochemicals, protecting the soil and reducing costs, while increasing the crop yield. Another example application is forest management. Forests and trees in general store carbon, retain water, contribute to the conservation of biodiversity and to public health. They help to mitigate climate change, and aesthetically support our well-being. Remote on-site monitoring of critical parameters of trees [1], illustrated in Figure 1, contributes to a better understanding and helps to predict the effects of climatic extremes on forests, urban trees and parks. The acquired information can provide crucial input for climate-smart and sustainable decisions [2].

Preventive conservation of cultural heritage can benefit from massive supervision of artworks and their surrounding conditions. A low-power IoT node was designed to monitor cultural heritage [3], and was deployed in the 17th century church of Santo Tomás y San Felipe Neri in Valencia, Spain. It enables micro-climate monitoring of the environment, in terms of temperature and humidity, to preserve the cultural heritage present in the church and the construction of the church itself. Key requirements were to realize a low-cost system to enable massive deployment, and a long battery life, that was estimated to reach a lifespan of over 10 years with a single AA Lithium-ion battery. Furthermore, it was important that the electronic devices could be embedded with as little aesthetic impact as possible, and that they could be installed by people with no particular technical skills. It should be noted that the connectivity is quite challenging as the radio waves need to penetrate thick walls and cover large distances, a goal that was achieved by operating on sub-GHz frequencies. Another customized system for monitoring the indoor climate was designed and installed in Adriana’s house in Pompeii, Italy [4]. The system sampled parameters every 30 min with 26 probes in 4 rooms for 372 days. Since the devices did not feature wireless connectivity, rather cumbersome manual retrieval of data was necessary.

Monitoring, waste management, and asset tracking in cities can contribute significantly to goals that these have set to become smart and sustainable, and improve the well-being of their inhabitants. The motivations for cities to embrace IoT technologies are diverse and stringent, and to meet these demands, various applications have been developed [5,6]. For instance, several solutions for remotely monitoring the status of garbage bins have been realized [7,8,9,10,11]. In particular to combat food waste, several IoT systems have been designed [12,13]. In the city of Suzhou, China, a solution encompassing edge devices, gateways, and cloud service, monitors the generation, collection, transportation and final disposal of restaurant food waste [13]. The IoT nodes have a reported lifetime of three years. In the context of improving sustainability and flexibility of mobility, shared bicycle providers are deploying IoT-enabled electronic locks and localization applications. Many other smart city applications have been realized, for example energy efficient solutions for air quality monitoring [14].

Industry 4.0 proposes a transformation to cyber manufacturing that heavily relies on connected sensors to increase efficiency and flexibility [15]. In this context, IoT solutions are deployed to optimize operation of a factory, ranging from predictive maintenance to asset tracking. Logistic warehouses are getting more automated, based on remotely controlled robots and IoT solutions to support the dynamism that is required to support the needs of on-demand shopping and e-commerce, as well as to increase overall efficiency [16].

### 1.2. Challenges in Connecting Remote IoT Nodes

**Figure 2:**
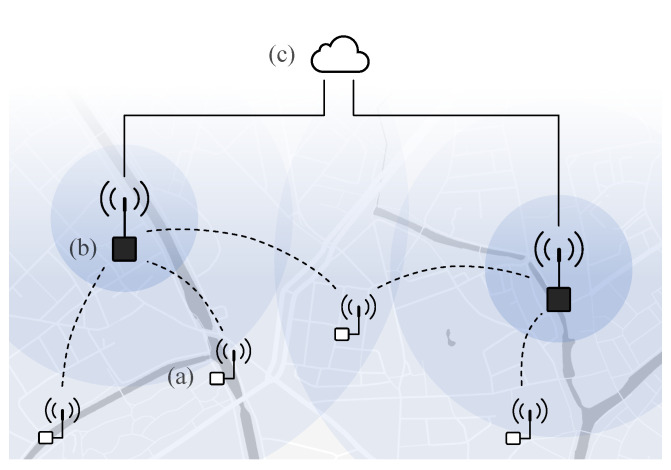
The typical architecture connects remote IoT nodes (**a**) via long-range connectivity to one of more gateways or base stations (**b**). The interconnected gateways relay the information to the server or cloud resources (**c**), where data can be visualized and interpreted.

While the above use cases serve a variety of application domains, they require very similar technological features and pose characteristic requirements to the overall IoT architecture sketched in Figure 2. This architecture comprises (a) the IoT devices that provide a sensing, measurement, and/or control functionality, (b) a wireless connection to a gateway or base station for information exchange with the remote device, and (c) a server or cloud platform where data can be stored, visualized, further processed and interpreted.

The desired characteristics of long-range IoT systems, raising technological challenges, can be found in three main categories and summarized as follows:

1. Remote autonomous operation: Ideally the nodes can be installed following a ‘deploy-and-forget’ approach, and are able to operate without short-term maintenance. They should be able to run on limited energy reserves, or on energy harvested from their environment. Ultimately, they will support self-management, including self-diagnostic capabilities. Many applications can tolerate that a packet of information gets delayed in delivery, or even sporadically gets lost.
2. ‘Light’ IoT nodes: The nodes should come with a low complexity and a small price tag, taking into account both hardware cost and communication plan. Their integration often requires a small form-factor. They mostly need to perform periodic sensor measurement (with a low duty cycle and non-time critical), with a-periodic time critical events. Intelligence at the gateway and/or cloud can be relied on, and a trade-off can be made in processing at the node or off-node.
3. Large area deployment: the nodes get deployed in a wide range of environments, in (sub-)urban as well as rural areas, and ranging from fields to forests. These applications require long-range wireless communication and have a low packet size. Many application require awareness of the location of the devices [13], while the devices themselves can operate more efficiently by exploiting knowledge on their context [17].

This paper focuses on how to connect and design remote IoT nodes and operate them as long as possible on a very restricted energy budget. It is essential to address challenges in all three categories mentioned above: (i) autonomy, (ii) low-cost and small form-factor, and (iii) large area deployment capabilities. To maximize the autonomy of long-range IoT devices, the energy budget needs to be managed meticulously, stingy, and correctly: creative design of the nodes is desirable, creative (energy) accounting is unacceptable. To estimate the expected energy need of IoT devices, the following approach can be taken:

1. Make an inventory of operational modes of the device, including active and idle states,
2. For each of the modes, estimate the power consumption of the node,
3. Calculate the energy consumption by weighing the power consumption with the expected time spent in these modes.

**Figure 3:**
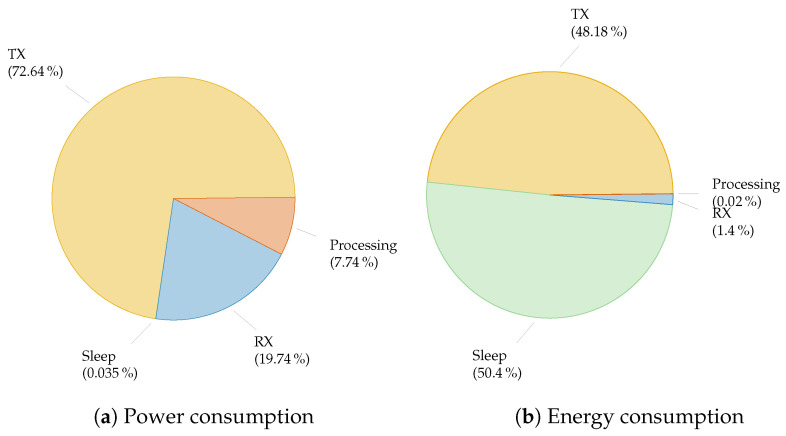
Power consumption and energy consumption share of each stage of a Long-Range Wide-Area Network (LoRaWAN) node. The energy corresponds to reading out a sensor and send ing a 16 bytes payload packet (SF12) every hour [18].

We illustrate this approach for a representative case in Figure 3. The results show, in the first place, that the wireless transmitter is one of the big spenders. IoT applications will require data to be sent from the device to the server, also called the uplink. This implies that the device needs to provide the power to the radio signals to carry the data and spend a relatively large time in this high power mode. This is in contrast to conventional mobile communication networks which are in general downlink dominated. Figure 3 also shows that, for sporadically active nodes, the sleep state has a high impact on the total energy budget, even if power consumption is very low in this mode, as this mode is dominating in time.

This paper provides insights in what causes battery drain of remote IoT devices, and proposes solutions to combat this. Selecting the right wireless technology is a first step in this process. Hence, we zoom in to the current state of the art Low-Power Wide-Area Network (LPWAN) technologies, in particular LoRaWAN, Sigfox and Narrowband IoT (NB-IoT). As demonstrated earlier, communication requires a large share of a node’s power consumption. As a consequence, battery preserving strategies such as, “think before you talk” and, “race to sleep” are advocated. These strategies, however, only pay off when combined with a careful hardware design. This design needs to ensure extremely low sleep power, because the consumption in sleep largely determines the nodes autonomy. To that end, we provide practical advise and illustrative examples that may help the embedded system designer. We further point out emerging technologies and promising R&D that may enhance the battery life of remote IoT nodes greatly in the future.

The remainder of this paper is organized as follows. In the next section we explain the inherent physical dilemma in establishing long-range wireless connectivity while keeping a low-power profile. We elaborate on approaches to overcome these, and sketch the landscape of available LPWAN technologies. In Section 3, we present the typical anatomy of an IoT node and we highlight the hardware design challenges to achieve a long autonomy. Next, in Section 4, we elaborate on key strategies to preserving battery life of remote IoT nodes. Finally, we summarize the conclusions of this overview paper in Section 5 and we indicate promising R&D directions that can progress remote IoT nodes to more and longer autonomy.

## 2. Low-Power Wide-Area Networks: The Technological Landscape

In this section we introduce the state of the art wireless communication technologies to connect remote IoT nodes. In particular, we focus on energy-constrained devices that require connectivity to the nearest access point or base station, at distances of hundreds of meters to tens of kilometers. Complementary to other overviews [19,20], this survey provides insights in transmission solutions to overcome the inherent discrepancy between low-power and a large coverage. We further assess the landscape of current IoT technologies, considering energy consumption in conjunction with communication characteristics.

The most prominent requirements for serving remote IoT nodes are long-range and low-power connections. Consequently, this paper focuses on Low-Power Wide-Area Networks (LPWANs) which are tailored to provide wide-area communication to power-constrained devices. LoRa [21] and Sigfox [22] are both LPWAN technologies which gained popularity in recent years and are now key players in this domain each bringing specific interesting features. These technologies operate in the unlicensed sub-GHz spectrum due to the favorable propagation characteristics needed for coverage extension. Furthermore, new cellular communication modes and terminal categories are defined, e.g., NB-IoT [23], for Machine-Type Communication and IoT applications. NB-IoT can operate both in sub-GHz and conventional cellular frequency bands. Notably, other technologies exist but are less adopted by the LPWAN vendors or are focused on higher throughput communication. For instance, two other cellular technologies are designed to support machine-type communication. EC-GSM extends the coverage of legacy Global System for Mobile Communications (GSM) but is less or not adopted by network vendors. In addition to NB-IoT, another technology based on Long Term Evolution (LTE) is specified, i.e., LTE-M. This standard, while less adopted than NB-IoT, also targets a different market, i.e., Machine-to-Machine (M2M). Furthermore, other technologies tailored for LPWAN communication such as RPMA, NB-Fi, Weightless, Wi-Fi Halow, DASH7 have seen less of an adoption by the LPWAN market and are therefore not further considered in this work.

Long-range and low-power connections require the co-design of both the physical [24] and medium access control layer [25]. The Physical (PHY) layer defines the modulation scheme applied to get bits of information transported by Electromagnetic (EM) waves. The Medium Access Control (MAC) layer specifies the manner in which the devices or nodes access the shared medium, i.e., the radio band. It coordinates the access to mitigate and minimize packet collisions and interference. An optimized design in both layers is required to achieve low-power operation. This section elaborates on the aspects by which the technologies address the low-power and long-range constraints. We first describe the possible design approaches at each layer. After which, the specific implementation of these approaches by each technology is further discussed. A summary of the design choices of each of these technologies can be found in Table 1.

### 2.1. How Can LPWAN Technologies Communicate on a Low-Energy Budget?

LPWAN technologies adopt simple yet adequate modulation schemes and energy-efficient MAC principles to support low-power operation. As illustrated in Figure 3, transmitting has a high impact on the energy consumption of the node. The energy required in the radio to transmit the information accounts for a share of almost 50% of the total energy consumption. Hence, this is addressed by LPWAN technologies to reduce the energy expenditure of an IoT node.

#### 2.1.1. Low-Energy Physical Layer

**Figure 4:**
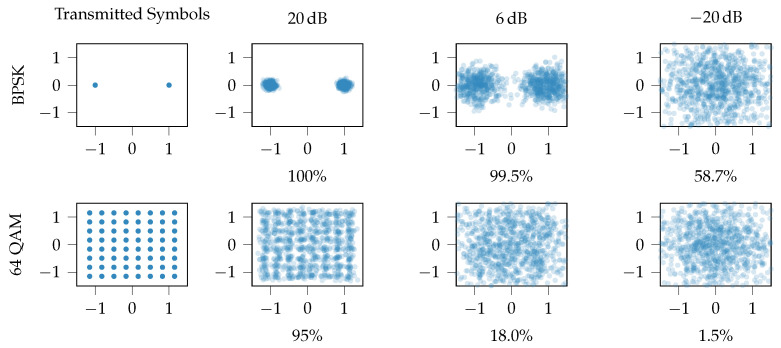
Constellation diagrams of BPSK and 64-QAM modulated symbols at different SNRs including the percentage of successfully demodulated symbols. It demonstrates that low-order modulation schemes are vital in order to work in low-SNR environments or when the transmit power levels are low.

Increasing the spectral efficiency was the main focus during the evolution of conventional communication technologies. Over different generations, going from 2G to 3G and 4G, this has been achieved by condensing the information per Hz. Information is digitally modulated and can be represented by constellation points on a complex plane, as shown in Figure 4 [26]. The more constellation points, the larger the alphabet of symbols that can be carried by a single sample. The length of this alphabet, i.e., the number of different symbols, is called the modulation order. To increase the spectral efficiency, higher order modulation schemes are used, densifying the constellation points. This makes these schemes more error prone than lower-order modulation, as additive noise at the receiver will provoke wrong decisions on which point constellation was actually transmitted. As a consequence, high throughput technologies require a high Signal-to-Noise Ratio (SNR), which is achieved either by transmitting over short links or with a significantly high power level. None of these are feasible for remote IoT devices. To tackle this, LPWAN technologies adopt more robust but less spectral efficient modulation schemes. The impact of the SNR on the constellation points is depicted in Figure 4. We illustrate the sensitivity to Additive White Gaussian Noise (AWGN) for Binary Phase Shift Keying (BPSK), transmitting one bit per symbol with a 2-symbol alphabet, and 64 Quadrature Amplitude Modulation (64QAM), transmitting six bits per symbol with a 64-symbol alphabet [26]. Here, we adopted typical SNR values [27] for long-range ( −20 dB at 4 km) and a short-range ( 6 dB at 20 m) connections. LTE (4G) defines a good quality link if the SNR is equal or higher than 20 dB. This is used as a baseline for comparison.

Figure 4 demonstrates why low-order modulation schemes are required for low SNR scenarios. Furthermore, the usage of low-order modulation schemes allows for less complex hardware, reducing the power consumption of the radio hardware. In particular for transmitting complex signals over a long range, the power amplification stage takes up more than 70% of the power budget, and achieves an efficiency of typically less than 30% because of the need to operate with significant back-off [28,29,30]. A power amplifier amplifies an input signal to a higher power signal. This amplification needs to be linear in order to not introduce distortions in the outputted signal. However, practical power amplifiers have a non-linear region close to their saturation point, causing distortions. The power level of the input signal is lowered to mitigate operating in this non-linear region. The higher the dynamic range of the input signal, i.e., the Peak-to-Average Power Ratio, the higher the required back-off. A PA is most efficient when working close to the saturation point. As a result, the PA is performing in a non-efficient manner due to the required back-off.

LPWAN technologies mitigate this inefficiency—in power consumption—by using constant envelope modulation schemes, where the amplitude of a signal is kept constant. To illustrate, Frequency-Shift Keying (FSK) transmits with an alphabet of symbols that have different discrete frequencies of their carrier signal [26]. This scheme does not alter the amplitude of the signal, relaxing the PA constraints. Furthermore, due to the reduced the data rate LPWAN technologies limit the payload size in order to lower the transmit duration. Besides a low payload size, the transmission power is also limited in the unlicensed bands–to respect the regulations.

##### Implementations in LPWAN

**Figure 5:**
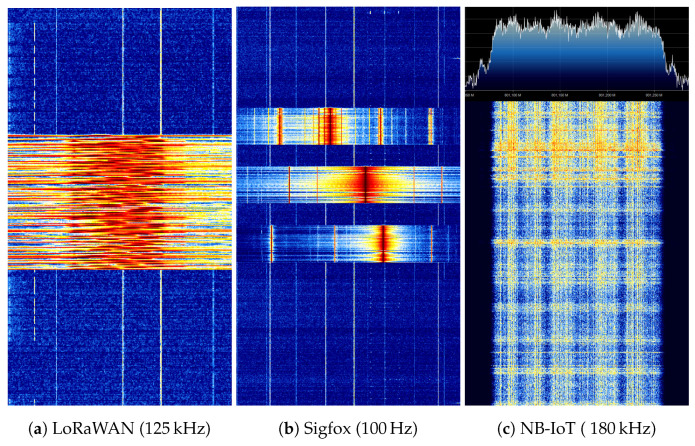
Measured waterfall spectrum of LoRaWAN, Sigfox and NB-IoT. The vertical axis represents the time and the horizontal axis the frequency domain. Due to the difference in time and frequency allocation, the figures have not the same time and frequency scale. The observed spectral leakage is due to the Fast Fourrier Transfomation (FFT) operation.

Each technology adopt different strategies, e.g., wideband vs. narrowband. This is illustrated in Figure 5 where a measured waterfall spectrum of a LoRaWAN, Sigfox and NB-IoT message is depicted. In LoRaWAN, Long Range (LoRa) and FSK are used at the PHY layer operating at 433 MHz and 868 MHz in Europe. Other frequency bands are used in different regions, please consult the frequency plan based on your region. FSK is used for short-range communication. The LoRa modulation scheme is based on Chirp Spread Spectrum (CSS). This technique encodes a single bit to multiple chirps. A chirp is a sinusoidal signal where the frequency is linearly increased or decreased in time over a fixed bandwidth. The spreading factor determines the duration of the chirps and hence the data rate. In other words, by increasing the spreading factor, the energy per bit is increased. This subsequently improves the SNR, yielding a higher sensitivity. The receiver sensitivity is a measure of the minimum required received Radio Frequency (RF) power to be received in order to be still able to demodulate the signal. A trade-off between range and energy consumption can be made by changing the spreading factor. Due to the additional gain by spreading the signal and having a constant amplitude envelope, inexpensive low-power high-efficiency PAs can be used.

In contrast to LoRaWAN, Sigfox uses an ultra narrow-band modulation scheme. Data is modulated by Differential Binary Phase Shift Keying (DBPSK) at 100 bps, generating a 100 Hz signal. Opposed to BPSK, where symbols are directly mapped to the constellation points, DBPSK modulates symbols by phase shifts. For example, a binary 1 results in a 180 ${}^{\circ }$ shift, while 0 does not introduce a phase shift. The system is more resilient against phase noise, as phase shifts are used rather than fixed constellation points. NB-IoT is mainly a slimmed-down version of LTE and reuses the modulation techniques employed in LTE [31]. In the uplink, NB-IoT uses BPSK or Quadrature Phase Shift Keying (QPSK) depending on the coverage [32]. The devices are multiplexed with Single-Carrier Frequency Division Multiple Access (SC-FDMA). In contrast to Orthogonal Frequency Division Multiple Access (OFDMA), SC-FDMA yields a lower Peak-to-Average Power Ratio (PAPR) [33] and thereby a lower back-off is required, resulting in a more power efficient PA operation. As a high share of the energy drain is a result of the transmission stage, in NB-IoT lower power classes are defined [23], i.e., 23 dBm (Power Class 3), 23 dBm (Power Class 5) and 14 dBm (Power Class 6). Power class 6 was recently introduced in 3GPP Release 14 [34]. These classes allow to simplify the PA and battery requirements thanks to the lower transmit power. While this brings a clear benefit for IoT devices, currently deployed networks are not prepared for these low-power transmissions and therefore these devices can be forced to employ a higher coverage extension, as further discussed in Section 2.2.

#### 2.1.2. Low-Energy MAC Layer

**Figure 6:**
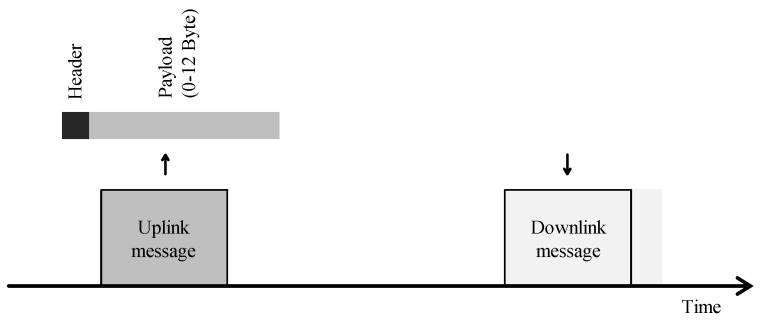
Typical MAC scheme for remote IoT devices. The communication is mostly device-induced with a limited payload and minimized protocol overhead. While downlink communication is supported, it is mostly restricted to a couple of messages per day.

Collisions, overhearing or idle listening, and overhead are the main causes of energy drainage related to the MAC layer [25]. Collisions occur when two nodes transmit and the signals overlap in time and frequency whereby the intended receiver is unable to demodulate the packet. Consequently, the energy consumed by both transmitter and receiver was wasted. Idle listening or overhearing is caused when a device is listening for downlink communication when there is none. Lastly, overhead due to additional signaling or protocol overhead introduces an extra energy drain. To address these issues, LPWAN technologies apply simple MAC schemes, as illustrated in Figure 6. The communication is mostly device-initiated, meaning that an uplink message is transmitted when the device has some data to send. The remainder of the time the device is kept in sleep mode. Overhearing and idle listening is particularly important in technologies operating in the unlicensed bands where everyone—respecting the regulations—can freely communicate. By specifying the start of a receive window, idle listening is kept to a minimum. Furthermore, no or limited signaling is used to request access to the network and the protocol overhead is constrained to the bare minimum in order to send small-sized packets over the network. As most applications allow for a packet loss to a certain degree, these protocols often follow a ‘fire-and-forget’ approach where collisions are of insignificant importance. As a result, the use of acknowledgments and retransmissions is less common and application-dependent.

##### Implementations in LPWAN

Both Sigfox and LoRaWAN operate in the unlicensed bands and follow a ‘fire-and-forget’ approach. They do not employ any multiple-access technique, i.e., ALOHA. They rely on collision avoidance techniques such as repetitions and narrow-band signals in Sigfox [35], and spread-spectrum in LoRaWAN to mitigate interference. Therefore, LoRaWAN and Sigfox are best effort LPWAN protocols without any guaranteed delivery. Notably, LoRaWAN supports acknowledgments and retransmissions to a certain degree.

**Figure 7:**
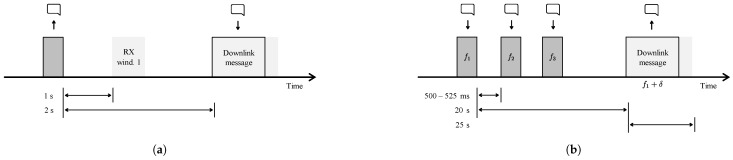
Medium Access Control mechanism for LoRaWAN and Sigfox. (**a**) LoRaWAN opens two receive windows after a transmit message. The first window utilizes the same data rate as the transmit message. The second window employs more robust settings to increase the chance of reception. (**b**) Sigfox transmits three duplicates at different frequencies and different time instances. Limited downlink is possible and is received at the initial transmit frequency plus an offset.

Moreover, both technologies specify the time and frequency of a receive window in order to limit idle listening. LoRaWAN (Figure 7a) opens a first receive window one second after the uplink message, where the downlink message has to use the same spreading factor and frequency of the transmitted packet. In case no preamble is detected, a second window is opened two seconds after receiving the uplink message at a default frequency and spreading factor. The default frequency (or channel) and spreading factor is 869.525 MHz and SF12 [21]. However, networks opt to use lower SFs in order to decrease the duration of the receive window. Both Casals et al. [18,36] have observed that requesting a retransmission can lower the overall energy consumption because the node does not open a second receive window after detecting a downlink message in the first window. For this purpose—and more extreme—Thoen et al. [1] propose to force the node in sleep mode without opening any receive windows. Similarly, Sigfox also supports bidirectional communication, while restricted to a maximum of four downlink messages per day. In contrast to LoRaWAN, Sigfox opens only one receive window (Figure 7b). After receiving a downlink message, the device responds with an uplink confirmation message [37].

**Figure 8:**
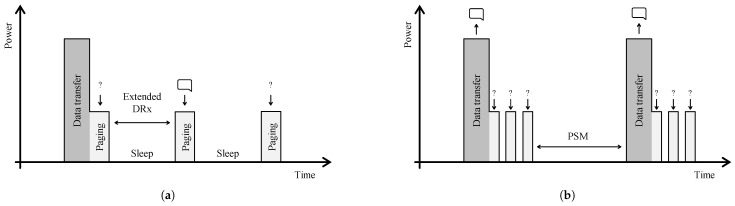
Energy saving strategies employed in NB-IoT, i.e., Extended Discontinuous Reception Mode and Power Saving Mode, respectively. (**a**) Listen more infrequently to downlink message with eDRX. (**b**) Hibernate between data transmissions with PSM.

NB-IoT, as a derivative of LTE, employs a more complex random access procedure [38]. The node first has to request access prior to transmitting an uplink message. The base station responds with a time and frequency slot where the node gets the opportunity to transmit. Furthermore, in contrast to the other technologies, NB-IoT is fully bidirectional, and not uplink-focused. Both node-terminated (paging) and node-induced traffic is supported, as illustrated in Figure 8. As paging and maintaining an active connection can be highly energy consuming, NB-IoT introduces Extended Discontinuous Reception Mode (eDRX) and Power Saving Mode (PSM). Both mechanisms are depicted in Figure 8. Extended Discontinuous Reception Mode allows a node to listening less frequently to downlink messages. These values are provider-specific, but they have to support at least an eDRX time of 40 min [39], compared to the maximum of 2.56 s in LTE. A higher energy reduction is achievable by employing PSM. In PSM the node notifies the network that it is going dormant and negotiates the duration of the hibernation. During this period, the node cannot receive any downlink messages but can transmit a packet without having to reconnect to the network. The maximum allowed time is 413 days [39]. In this manner, applications sending one packet per day can configure a PSM of 1 day. In case a critical message has to be sent, the node can initiate a transmission without having to wait till the end of the agreed-on hibernation period.

Prior to transmitting messages, nodes have to join a network. This initial access overhead is minimized in LPWAN. Sigfox does not use any joining procedure, while LoRaWAN supports both non-initial access and initial access protocols, i.e., Authentication By Personalisation (ABP) and Over The Air Authentication (OTAA), respectively. In NB-IoT, a node has to register to the network after a power-down. Fortunately, through PSM a node does not have to re-join to the network after a wake-up.

### 2.2. How Can LPWAN Technologies Achieve a Long-Range Connection?

LPWAN technologies extend the communication range also by a co-design of PHY and MAC layer approaches: (1) utilizing low frequency bands with favorable propagation characteristics, (2) applying modulation schemes which are less sensitive to a low SNR, (3) adopting MAC schemes to increase the probability of successfully receiving a packet and (4) dynamically adapting to the link conditions.

#### 2.2.1. Long-Range Physical Layer

In order to extend the range, LPWAN technologies utilize sub-GHz frequencies. The benefits of lowering the carrier frequency is illustrated by the Friis transmission Equation (1) [40]. The received and transmit power is denoted by $P_{r}$ and $P_{t}$, respectively. The directivity of the antenna is represented by *D*. The directivity of an antenna is angle-dependent. Hence, the directivity in the Friis formula depicts the directivity in the direction of the other antenna. The distance between the transmitter and receiver is denoted by *d*. The wavelength of the signal is expressed by λ. In other work, the antenna gain (*G*) is sometimes used instead of the directivity (*D*). As this expression is defined for ideal scenarios both expressions are equivalent as we have an antenna efficiency ϵ of 1 and G=ϵD. The formula demonstrates that the received power ($P_{r}$) is proportional to the square of the wavelength (λ). To be complete, the received power depends on the effective area or aperture of the antenna. This area describes the amount of power captured from the EM wave impeding on the antenna and is depending on the wavelength. It shows that more energy is transferred at lower frequencies due to the larger antenna aperture. Therefore, the path loss, defined as the ratio of the received power to the transmitted power, is also dependent on the wavelength. While this formula is defined for ideal free-space conditions, this frequency-depended path loss also holds in real environments. Moreover, objects appear smaller and have less impact of the signals at lower frequencies because the wireless propagation of the signals is affected by the objects relative to the signal’s wavelength.

(1) $$\frac{P_{r}}{P_{t}}=D_{t}D_{r}{\left(\frac{\lambda }{4\pi d}\right)}^{2}$$

As illustrated in Figure 4, low-complexity modulation is better suited for low SNR scenarios. In addition, the transmit power of the nodes can be lowered in case the SNR is higher than required. In other words, reducing the data rate increases the sensitivity of the receiver and thereby extends the coverage.

##### Implementations in LPWAN

LoRaWAN, Sigfox and NB-IoT operate at lower frequencies to benefit from the favorable propagation and adopt low-order modulation schemes. The range can be extended in LoRaWAN by increasing the spreading factor, thereby lowering the demodulation floor. For example, with a spreading factor of 12 a signal with an SNR above −20 dB can be demodulated, while for SF7 a minimum SNR of −7.5 dB is required in order to demodulate the packet. NB-IoT on the other hand switches between demodulation techniques, i.e., BPSK and QPSK, depending on the link condition.

#### 2.2.2. Long-Range MAC Layer

LPWAN technologies use diversity techniques to increase the probability of successfully receiving a packet: time, spatial and frequency diversity. In other words, by transmitting on different frequencies, at different time instances and by using multiple receivers, the probability that a packet is lost is lowered. To further increase the probability of successful receiving a packet, the PHY layer is controlled and adapted to the current link conditions.

##### Implementations in LPWAN

As an instantiation of applied diversity techniques, Sigfox sends three duplicates of the same packet, as depicted in Figure 7. Each packet is sent on another frequency at another time instance. Sigfox operators also try to densify the network so that all packets are received by at least three gateways. In contrast to Sigfox, LoRaWAN and NB-IoT employ mechanisms to adapt to the channel conditions. In NB-IoT three CE levels are defined, i.e., normal, robust and extreme. In the highest CE level (CE level 2) 128 repetitions, a higher power class 23 dBm and single tone transmissions are utilized to extend the coverage. The maximal tolerable loss or Maximum Coupling Loss (MCL) is defined for each of these coverage levels, i.e., 144 dB, 154 dB, 164 dB. Equivalently, in LoRaWAN the ADR algorithm [41] alters the spreading factor and transmit power to accommodate for more losses in channel. Furthermore, different spreading factors yield orthogonal packets, e.g., a SF7-modulated packet is not interfered by a SF12-modulated packet. By adopting ADR, the utilized spreading factor in the network is optimized, thereby reducing the number of collisions.

## 3. Anatomy of an Internet of Things Node

**Figure 9:**
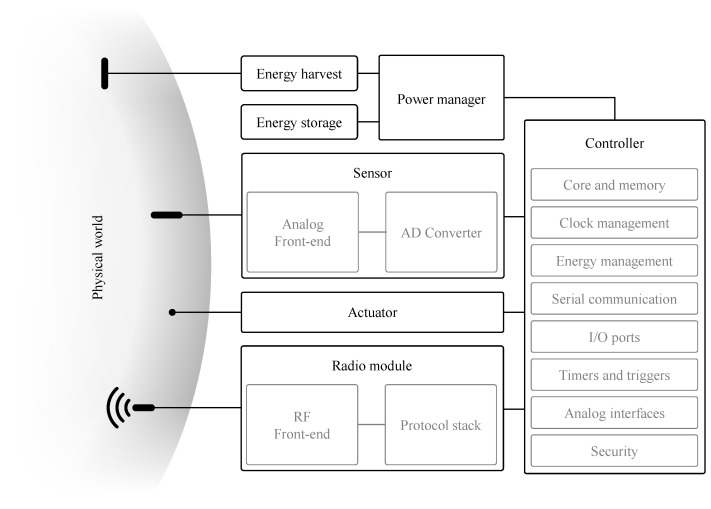
Generalized architecture of an IoT node. The complete device is typically battery-powered, optionally assisted by some kind of energy harvesting technology, e.g., a solar panel. Sensors periodically sample the environment for information, which is transmitted wirelessly. A microcontroller has all the amenities to manage the operation and behavior of the node. Actuators, e.g., a LED, allow the node to influence its environment or signal the user.

The typical anatomy of an IoT device, and its building blocks, is shown in Figure 9. It consists of a controller, a wireless radio module, sensor(s) and an energy source. In the following paragraphs we discuss each of these components and highlight the important properties that are needed for IoT devices to operate while using as little energy as possible. The strategies to achieve this, are discussed in Section 4.

### 3.1. Controller

The IoT node’s operation is controlled by the microcontroller by executing embedded software, also referred to as firmware. Core clock frequencies can range in order of magnitude from 10 kHz to 100 MHz. The clock frequency affects both processing speed and power consumption (see Section 4 for more details). Control over the node’s embedded hardware is facilitated by the controller’s broad range of built-in peripherals. General-Purpose Input/Output (GPIO) allows for driving basic digital signals, e.g., enable/disable subsystems, as well as reading from them, e.g., status indicators. Hardware support for digital serial protocols [42], such as, Serial Peripheral Interface (SPI), Inter-Integrated Circuit (I2C) and RS232 help with interfacing with subsystems such as, the radio module or the sensors.

Equivalent to the digital communication protocols, analog interfaces are present on a controller to interface with analog sensors. An important example is the built-in Analog-to-Digital Converter (ADC), which omits the need to include an external ADC on the node’s Printed Circuit Board (PCB). To secure the radio communication, dedicated security blocks are often integrated in the controller enabling acceleration of encryption algorithms. For example, Silabs’ EFM32^TM^ Arm Cortex-based line of microcontrollers integrate hardware acceleration, among others, for AES, SHA and ECC. Timers, whether or not independently clocked, allow for time-keeping and periodic event firing, e.g., to periodically initiate a sensor measurement. Finally, several features specifically aid in energy management of the controller itself. Power for different subsystems can be enabled or disabled and the clock speed can be throttled. The core itself can be put in sleep, while some peripherals remain active. The controller’s interrupt system can wake-up the core when an event occurs, e.g., A timer fires, or even initiate data transfer between two peripherals, bypassing the core altogether. This is the so-called Peripheral Reflex System (PRS) system, a network that lets the different peripheral modules communicate directly with each other without involving the core [43].

### 3.2. Sensor

A rich variety of phenomena and signals can be observed using a broad selection of sensors, using different operating principles [44]. Today, the choice is nearly unlimited. Several selection criteria can be applied when choosing a sensor with a certain application in mind. Common functional specifications such as supply voltage and required PCB real-estate are highly application dependent. Choosing a sensor that is able to operate on the same supply rail as the microcontroller, generally simplifies the interfacing between the two.

Digital versus analog. In cases where raw, unprocessed sampling is required, an analog sensor is to be preferred over a digital sensor. Moreover, when ultra-low power operation is required, an analog solution can be advantageous. However, the design of the analog front-end that is responsible for signal conditioning, e.g., amplification or filtering, increases development time, and creates new challenges in terms of power management. Digital sensors, typically connected to the controller using I2C or SPI [42], can simplify the hardware design process significantly. Multiple sensors can be connected to the same I2C-bus, effectively saving pins on the microcontroller. Sensing element, analog front-end and additional logic circuits are integrated on the same device. When the sensor readings are (pre-)processed on-chip before communicating with the microcontroller, it is referred to as a smart sensor [45]. A typical and quite handy example is an Inertial Measurement Unit (IMU). For example, the ADIS16488A IMU is a complete inertial system that includes gyroscope, accelerometer, magnetometer, and pressure sensor readings. The factory calibration characterizes each sensor individually and programs it with its own dynamic compensation formulas in order to provide accurate sensor measurements [46].

When selecting a digital sensor, a type with certain integrated features is preferable, as these features will help to implement some energy-saving strategies (see Section 4). Examples of such features are:

- A sleep or power-down function simplifies power management,
- Automatic sampling (with programmable period) omits the need for extra communication with the microcontroller, i.e., it does not continuously need to “tell” the sensor to start a new measurement,
- Programmable thresholds can further reduce communication between the sensor and the controller. For example, an accelerometer will only signal the controller when the measured acceleration is above a certain G-force.

Keep in mind that, to facilitate these smart functions, additional signals are often required. For example, an alert pin on a sensor (indicating that measurements have exceeded a set threshold), needs to be connected to one of the microcontroller’s inputs.

What and how to measure? While some applications require only measurements of a single parameter, many, e.g., environmental monitoring, involve the collection of several parameters. It is common to find sensors that integrate a set of related measurements. For example, the BME680 is a digital low-power gas sensor that is able to measure air temperature, humidity and pressure, specifically suited for indoor operation [47]. Furthermore, it is able to detect Voltatile Organic Compounds (VOCs), making it suitable as an (indoor) air quality sensor.

Quite often applications need knowledge of where the measurement(s) take place. If sensor nodes are mobile, or if deployment is random, e.g., by an airdrop, the position of the sensor is a measurement in its own right. To locate an IoT node, an obvious course of action is to incorporate a Global Navigation Satellite System (GNSS) receiver into the device. A GNSS receiver can locate the node by using one of the GNSS satellite systems (e.g., Global Positioning System (GPS) or Galileo). The module can compute a real-time position using satellite information: latitude, longitude and altitude within a radios of 10 m or better [48]. However, localization comes with an non-negligible energy cost due to the long setup time. More energy-efficient approaches exist by using native geolocalisation based on the uplink messages. The advantage of this approach is that this information comes “free”, i.e., it can be extracted from the communication needed to send the sensor measurements. This is further discussed in Section 4.2.2.

### 3.3. Radio Module

**Figure 10:**
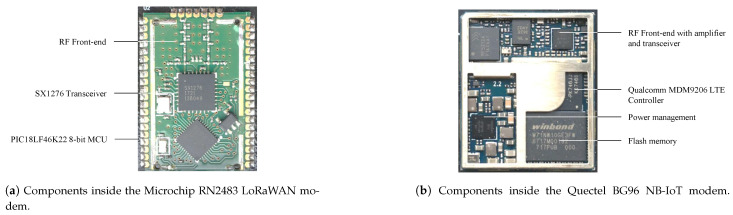
Modems combine RF front-end, transceiver and MAC layer implementation on a controller in one easy to use module. These examples show the internal components of two popular IoT modules. While the building blocks of these modules are independent of their manufacturer, these modules are chosen because of their high availability and adoption in IoT nodes.

The radio module is responsible for exchanging information wirelessly. Depending on the IoT technology, two options are available, i.e., a transceiver or a modem (Figure 10). The former supports only the PHY layer of the protocol. Often, the RF front-end, including also the PA, is integrated on chip. The MAC layer needs to be implemented on the controller. A (wireless) modem, on the other hand, simplifies interfacing by implementing both the PHY and MAC layer. A modem is typically controlled by means of ATtention (AT) commands. The benefit of using transceivers, is that only one microcontroller is present on the device, while the modem also incorporates a controller to handle the MAC. Although these embedded controllers are also ultra low-power, it still yields a non-negligible energy penalty, as can be observed in Table 2. Despite this, modems significantly lower the development effort as no MAC layer protocol has to be implemented on the microcontroller. This also ensures that the node respects the protocol specification. Both approaches are sometimes combined where the microcontroller—in the System on Chip (SOC)—can be directly programmed with user-specific code. For example the Nordic NRF52832 allows to program the internal Arm while still retaining the protocol stack.

### 3.4. Power Management

Power management on the IoT node falls into two main parts. A first part is the voltage regulation, which ensures that the voltage input, either from the battery, energy harvesting or any other power source, is converted into the operating voltage for the node’s electronics. Most often this includes a Low-Dropout Regulator (LDO) voltage converter or switching mode voltage regulator. Please note that these electronics might require different operating voltages. In any case, these voltages are distributed to the controller and all peripherals through the so-called supply rails.

**Figure 11:**
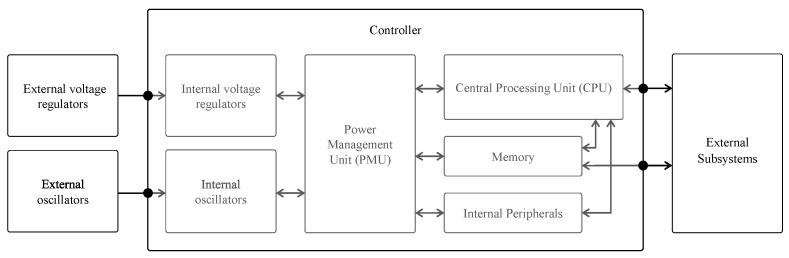
The internal Power Management Unit (PMU) of a microcontroller supervises other Microcontroller Unit (MCU) building blocks. Depending on the current controller state, different voltage regulators are connected or disconnected. The firmware on the CPU can, in turn, enable or disable external subsystem, such as, sensors or the modem.

A second part is the power switching, which is typically governed by the firmware running on the Central Processing Unit (CPU) (see Figure 11 for the blocks involved). The power switching, in turn, falls into two parts. Firstly, the external subsystems, such as sensors, modem or even complete supply rails, can be enabled or disabled by the CPU. This typically involves load switches or control signals that are connected to the controller’s GPIO. Of course, such type of control requires that the appropriate measures are taken when designing the hardware (see Section 4.1.2).

The power inside the controller is managed through the PMU. One task of the PMU is providing a constant supply voltage to components inside the controller. Another task of the PMU is to control power distribution inside the controller, i.e., cut-off or provide power to certain blocks. By doing this, different power states are supported each providing different features. For the well-known Arm-based types of microcontrollers, that are much-used in IoT designs, these are the so-called energy modes.

### 3.5. Energy Storage

Most remote IoT nodes rely on batteries as an energy storage and provisioning solution. The most commonly used battery technologies are discussed in this section, summarizing their main relevant characteristics in view of the specific requirements of IoT devices. Other energy storage technologies exist but are less suitable for IoT devices. For example, current state of the art Electric Double-Layer Capacitors (EDLCs) have a high self-discharge yielding this technology unsuitable for long-lived applications. Rechargeable batteries, on the other hand, are especially suited in combination with energy harvesting techniques. Lithium Cobalt Oxide (LCO) and Lithium Polymer (LIPO) batteries are currently the most used rechargeable technologies. The attractiveness of such batteries is limited mostly due to their constrained operating temperature, as their capacity is severely impacted by the temperature. In addition, LCO and LIPO have a higher self-discharge rate compared to non-rechargeable batteries. This self-discharge limits the autonomy of IoT devices. Non-rechargeable batteries, while being one time usable only, have some favorable properties compared to the above rechargeable batteries, not in the least their self-discharge rate. The generally higher energy density and operating temperature makes them more suitable for deploy-and-forget IoT applications. To support a long operating time, the considered battery technologies have a capacity in the order of several ampere hours. Battery technologies such as Silver oxide, Zinc Air and Zinc Cloride, typically packaged in small button cells, have a lower capacity [49] and are therefore not included in this study.

The various parameters that are vital for remote IoT nodes are discussed below and grouped by their impact. Table 3 shows various energy storage technologies and their characteristics based on the manuals of corresponding battery compositions. By selecting the most optimal battery technology, the node’s operation time is maximized. As a result, the batteries of these devices require less battery changes and hence less maintenance, further reducing the impact on the environment.

#### 3.5.1. Energy and Power Density

The energy density of a battery determines the weight or volume required for a certain desired capacity. Applications requiring a small form-factor, require a high volumetric energy or weight energy density storage technology. To support possible high-power applications, high power density batteries are better suited for nodes with actuators such as motors inducing power peaks. Despite this, IoT devices are generally not meant for high-power applications.

#### 3.5.2. Battery Discharge

Due to internal chemical reactions, a battery loses charge over time even when no load is connected. This self-discharge rate determines the shell life of the battery and reduces the autonomy of the node. Non-rechargeable batteries mostly have self-discharge rates lower then 3% per year. Rechargeable batteries usually have a self-discharge rate above 10% per year [57,58]. They usually have a safety circuit causing an additionally capacity decrease of a few percent per month. When possible, non-rechargeable batteries are preferred in low-power IoT applications because of the self-discharge rate. Ultimately, this will be the limiting factor and possibly determine the autonomy of an ultra low-power IoT node.

#### 3.5.3. Energy Capacity

The energy capacity that can be extracted from an energy source by an IoT node, is limited by both the voltage range and the discharge rate. The discharge cut-off voltage is the lowest value in the voltage range of the battery: no more power can be delivered, the battery is considered empty. However, the controller and other peripheral electronics of the IoT node, may not be able to cope with such low voltages. The remaining battery capacity, will therefore be lost.

The nominal voltage and in addition the entire battery voltage range determine the external power management implementation. If the maximum and minimum voltages of the energy storage technology are within the voltage range of the MCU and peripherals, no additional voltage conversion is technically required. Considering the general voltage range of controllers: 1.98–3.8 V. When an EDLC is used (typical voltage range of 0–2.7 V), a voltage converter to boost the voltage will be required to optimally use the capacity of the battery. When a series of three Alkaline batteries (typical voltage range of 2.7–4.5 V) are used, however, the voltage will need to be lowered for the maximum voltage to fit inside the controller voltage range. This can be achieved by implementing an LDO voltage converter or a step down switching voltage converter.

Faster discharge rates result in more internal losses, voltage drops, and a lower usable battery capacity. Therefore, the maximum recommended discharge rate will be defined by the manufacturers. For most battery technologies, IoT nodes will not exceed this limit.

#### 3.5.4. Operating Temperature

Depending on the temperature range and conditions the nodes will be deployed in, the operating temperature of the battery may be an important characteristic. While the self-discharge generally will be lower at low temperature, the battery capacity and cell voltage will decrease. Lithium Thionyl Chloride (LTC) non-rechargeable batteries can operate in a wide temperature range and are most resistant to extremely low temperatures–down to −55 ${}^{\circ }C$. Only high loads <250 Ω and extremely low temperatures −18 ${}^{\circ }C$ reduce the battery capacity sharply [59]. The capacity of Lithium Manganese Dioxide (LMD) and Lithium Iron Disulfide (LID) batteries reduces by, respectively 35% and 20% at temperatures lower than −20 ${}^{\circ }C$ [52,53]. Alkaline batteries are more sensitive to lower temperatures. A recommended operating temperature range is −18 to 55 ${}^{\circ }C$. The capacity decreases from ∼2.5 Ah (20 ${}^{\circ }$C) to less than 0.5 Ah ( −20 ${}^{\circ }C$) [60]. Concerning LCO batteries in cold conditions, the state of charge can drop from 100% to 77%, if the cell temperature decreases to −15 ${}^{\circ }C$. The cell voltage of both LIPO and LCO batteries drops sharply with temperature. The voltage, of a full charged LCO battery, can drop from 4.2 V at room temperature to 3.8 V at −15 ${}^{\circ }C$ [61].

Generally, the discussed non-rechargeable batteries have a shelf life of 10 years that can decrease significantly due to bad storage conditions. For example, the shelf life of Alkaline (ALK) batteries is the longest at temperatures of 0 ${}^{\circ }C$ and is typically 10 years at 20 ${}^{\circ }C$. Higher temperatures results in a higher self-discharge, for example, a remaining capacity of <80% after 5 year when operating or keeping the battery at 40 ${}^{\circ }C$ [60].

#### 3.5.5. Alternative Rechargeable Storage Solutions

Devices with an energy harvesting system require a rechargeable energy storage solution. Beside the two mentioned battery technologies (LCO and LIPO), a.o. EDLC, Lithium-ion Capacitor (LIC), and Lithium Titanium Oxide (LTO) storage technologies are eligible. If an IoT application allows a battery technology with a lower volumetric energy density, meaning a low energy need and/or relatively large space is available, an LIC or an LTO solution may qualify. These two solutions have a high cycle life, and as they can be recharged often they can last long. Such a solution results in a small ecological footprint. LIC batteries have a higher volumetric energy density and a lower self-discharge compared with EDLC. 30 days after a full charge, the open circuit voltage of an LIC still remains 94% which is much higher than of an EDLC 79.6% [62]. LTO batteries also have an acceptable self discharge. 30 days after a full charge, the capacity remains 97.77% [63]. The LIC and LTO have additionally a low internal resistance, high power density, high safety level and an operating voltage range, respectively 2.2–3.8 V and 1.9–2.8 V [64,65].

In conclusion, several aspects need to be considered when selecting a battery technology and there currently does not exist one that is superior in all conditions. The first question to answer is whether a rechargeable solution is needed or not. Next, energy and other electrical requirements and application constraints (e.g., in terms of available space and operating temperature) need to be considered. Of particular importance for remote IoT devices that are performing only sporadic measurements and have no energy harvesting capabilities, is to select a solution with low self-discharge. Indeed if one wants to leave the device without maintenance for many months or several years, this parameter may be determining the actual autonomy of the device.

### 3.6. Energy Harvesting

**Figure 12:**
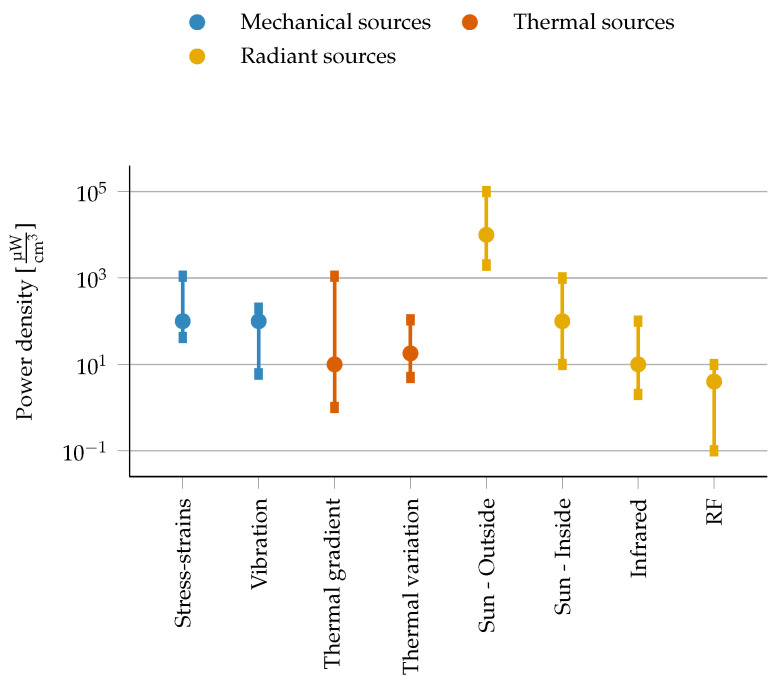
The potential power density in function of different energy harvesting approaches is shown, with the actual produce heavily depending on the stimulus. A distinction is made between three energy harvesting sources: mechanical, thermal and radiant [77,80,81].

Energy harvesting facilitates energy provision during deployment, prolonging the autonomy of devices—theoretically even to an unlimited time. Several sources of energy can be harvested from, either in the ambient environment or from external sources. Ambient sources are available/in the air spontaneously, while external sources of energy are deliberately delivered to devices. Recently energy harvesting solutions are also designed specifically for small IoT devices [10,66]. We here only provide brief comments on the scala of technologies, and refer the readers to the many publications that provide an overview of technologies [67,68,69]. Typical values for energy harvesting sources such as solar, vibration, thermoelectric and radio frequency energy are depicted in Figure 12. Please note that this provides an indicative relation only, as a power density is not a straightforward metrics for all the considered technologies. The employed energy harvesting technique will primarily be determined by the application itself and the environment where it is deployed.

#### 3.6.1. Sun and Light Sources

Light sources are providing energy via radiation that can be harvested based on the Photovoltaic (PV) effect. Solar power harvested through PV systems by far generates the largest amount of energy [70] of the different harvesting techniques, foremost in outdoor scenarios, generating a density that is about two orders of magnitudes higher then other sources. Organic PV systems can be integrated in flexible shapes and offer further advantages in producing less toxic waste, at the expense of lower energy harvesting efficiency [71]. PV systems operating indoors on indirect natural or artificial light. Their efficiency will typically be orders of magnitude lower than outdoors, and will clearly drop drastically in basements or rooms that are hardly illuminated. Solar energy is also a less attractive source of energy for small form-factor devices, although dedicated implementations of PV solutions for IoT nodes have been reported [66]. A solar energy harvesting system generally consists of a combination of a solar panel, an Maximum Power Point Tracking (MPPT) implementation and a DC/DC converter [72] to charge a battery. Infrared radiation is an energy harvesting method that powers mostly bio-implantable devices [73], where an external source is used to supply the infrared radiation.

#### 3.6.2. Radio Frequency Harvesting

RF signals offer the possibility to remotely power devices. This technique is particularly promising in terms of convenience [69,74]. Energy harvesting via RF requires an RF to DC converter, a DC/DC converter and mostly also a MPPT controller. Through a DC/DC converter, a battery or capacitor can be charged providing energy to the IoT device. The amount of received RF energy depends on many parameters, including transmitted power, the gain of the receiving and transmitting antenna, the frequency of the transmitted RF signal, the distance between the RF transmitter and the efficiency of the RF Harvester. The distance between transmitter and receiver is generally large for devices located outside. The received power will be small, and is thus infeasible to be used for energy harvesting [69]. A specifically interesting approach to exploit this technique is to maximize sleep time of wireless connected devices by using wake-up radios [75,76].

#### 3.6.3. Mechanical Energy Harvesting from Vibrations

This technique converts vibration into electrical energy. A relative movement between two elements is required for the conversion. A mechanical to electrical converter generates voltage. This could be a piezoelectric material, magnetic coil or variable capacitor. The generated voltage is usually low. An additional spring can cause resonance, increasing the amplitude and thus increasing the harvested energy [77]. The harvesting technique from vibrations can hence only be considered for specific applications where these are inherently present, such as for example in a Tire-Pressure Monitoring System (TPMS). MPPT system is recommended to increase the power extraction of the source and thereby the efficiency of the power transfer. The implementation depends on the energy harvesting technology. Several MPPT techniques exist to achieve the highest efficiency. A trade off between cost and efficiency has to be made [78].

#### 3.6.4. Thermal Sources

Thermoelectric energy harvesting requires a temperature difference between junctions in a solid-state device that operates based on the Seebeck effect to covert a thermal difference into a voltage. This technology generates energy from body or solar heat and can power medical devices or implants, personal wireless networks or other consumer devices. A thermoelectric generator ensures the thermal-to-energy conversion. Acceptable temperature differences mainly occur in body IoT applications. Temperature differences in outdoor IoT appliances are usually smaller. An example is the Seiko Thermic watch producing 22 μW of power (at 300 mV) with a temperature difference of 1.5 ${}^{\circ }C$ over the thermoelectric energy harvester [79].

In conclusion, the application itself will be determining whether, and if so which type, energy harvesting can be relied on. Solar energy harvesting is by far the most viable option for remote IoT applications in outdoor environments, when the related cost and size are affordable. Other options will mostly be insufficient for size-constrained IoT devices that need to support long-range connectivity.

## 4. Strategies for a Long Battery Life

In the previous section, the building blocks of an IoT node were discussed. In this section, the exploitation of feature-sets and component selection in combination with tailored software design for low-power operation is elaborated. While in Section 2 the energy impact of transmission is considered, the energy consumed in sleep is equally important as evidenced in Figure 3 and is studied in this section. Furthermore, an IoT end-device needs to meet the specific requirements of the considered application, as pointed out by the diverse use cases introduced in Section 1. This obviously has a major impact on both the hardware and software design. Not only will choosing the right components help a long way towards a small form factor and reduced energy consumption, it will also allow to apply certain energy saving strategies to get even more from your battery.

**Figure 13:**
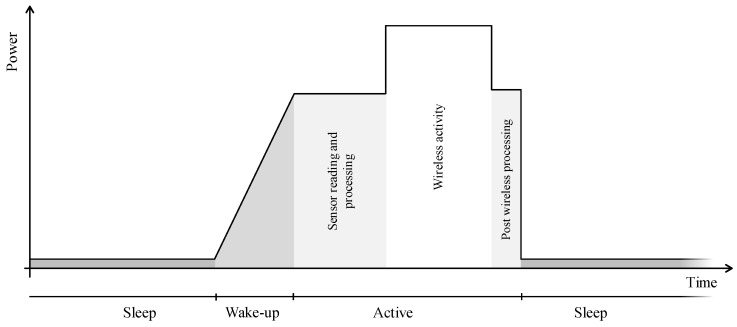
Typical power profile of an IoT node. This periodically recurring profile generally consists of four states, i.e., sleep, wake-up, processing and connectivity. A node typically spends most of its time in the sleep mode. The area under the curve yields the energy consumed in each state.

A typical power profile of a sensing IoT node is depicted in Figure 13. This periodic profile generally consists of four states: sleep, wake-up, processing and connectivity. A node should spend most of its time in the sleep mode: the lowest possible power state. Several monitoring systems can remain active in this state, but no processing or wireless communications are possible. When an event wakes the node, the controller core and peripherals are woken, which is followed by a transition period where components, such as analog circuits and oscillators, have to become stable before use. When this period is completed, the node can enter the ‘active’ state. In this state, the necessary data is gathered and prepared for wireless transmission. After all wireless communication is completed, the node might do some additional processing, e.g., reconfigure a sensor’s settings based on the received downlink message, and goes back into sleep mode. Note, that the wireless activity is optional and is not always required—or even desirable—in each cycle, as discussed in Section 4.3.2.

The total energy consumed in one cycle can be noted as the sum of the energy used in each state.

(2) $$\begin{array}{cc} E_{\mathrm{total}} & =E_{\mathrm{sleep}}+E_{\mathrm{wake}-\mathrm{up}}+E_{\mathrm{active}} \end{array}$$

It is clear that to save energy, the power consumed in each of these states should be minimized. More importantly, high power states (such as wake or active) should be strictly limited in time. This allows for more time in low-power states (sleep) and, hence, leads to energy savings. In the following subsections we discuss possible energy saving mechanisms in each node state, i.e., sleep, wake-up and active. Note that the node’s energy modes do not correspond to the Arm Energy Modes, as it includes power consumed by sensors and wireless communication. However, Arm Energy Modes help to keep the node’s power consumption as low as possible in any given mode.

### 4.1. Sleep

**Figure 14:**
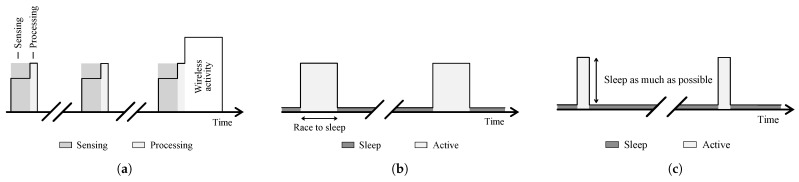
Proposed energy saving strategies. The applicable strategies—or combination of strategies—strongly depends on the application. (**a**) Think before talk. Check the validity of the sensor measurement and optionally accumulate data prior to transmission. Some sensors do not require the MCU to be active yielding a lower power consumption as depicted by the border. (**b**) Race to sleep. Utilize dedicated hardware features to reduce time spend in high-power states. (**c**) Sleep as much as possible. Avoid powering unnecessary hardware and fall-back to a sleep state as often as possible.

We suggest to follow the rules “sleep as much as possible” and “race to sleep”. Through the former approach (Figure 14c), other high-power states should be avoided as much as possible. Despite the possibility of a higher power consumption, the latter (Figure 14b) proposes to consider speeding-up the microcontroller to more quickly enter the sleep state. Furthermore, hardware accelerated blocks can further decrease the processing time and hence, increase the time spend in sleep.

By limiting the time spent in active mode to the absolute minimum, the device spends the majority of its operation in sleep mode. Therefore, the energy spent in sleep mode is often a dominant parameter in the overall energy consumption (Figure 3). To save energy, all peripherals and unnecessary components are powered down. To trigger the controller to wake-up, several mechanisms can be used, i.e., external interrupts, Real Time Clock (RTC) or a watchdog timer. In this part, the impact of the controller, PMU, sensors and the radio module on the sleep energy is studied. Based on this, we propose strategies to reduce the energy expenditure in sleep.

#### 4.1.1. Controller

The power consumption in modern microcontrollers—and any digital Complementary Metal–Oxide–Semiconductor (CMOS) logic—is mainly determined by the switching power ($P_{\mathrm{switch}}$) and static power consumption ($P_{\mathrm{static}}$), as illustrated in Equation (3) [82]. Switching or active power refers to the power consumption due to the switching of the digital logic. It depends on the supply voltage $U_{\mathrm{DD}}$, the CMOS switching activity factor α, the load capacitance $C_{L}$ and the frequency *f*. In other words, the dynamic power depends on the number of transistors, changing state per cycle, and the core clock speed. In other words, if more peripherals are being clocked or a higher clock speed is used, power consumption will also increase. In addition, the supply voltage will have a high impact on both the static and the active power consumption. Hence, lowering the supply voltage is sometimes used to further decrease the power consumption as discussed later-on.

(3) $$\begin{array}{cc} P_{\mathrm{avg}} & =P_{\mathrm{switch}}+P_{\mathrm{static}}\\ \; & =\alpha C_{L}U_{\mathrm{DD}}^{2}f+I_{\mathrm{leak}}U_{\mathrm{DD}} \end{array}$$

However, in sleep mode, almost all clock sources are disabled and thus there is almost no logic active. Only static power remains in sleep mode [83]. Static power refers to CMOS leakage currents. Leakage current will be the most dominant factor during sleep and is primarily caused by the output stage, i.e., Input/Output (IO) pins. For example, a 30-pin input device which has a leakage current specification of 100 nA, can easily consume up to 3 μA while in sleep mode [84]. To minimize leakage currents, controllers incorporate an advanced PMU enabling ultra-low power sleep currents. The PMU will cut supply to any unnecessary logic modules during sleep. Peripherals such as flash, Random-Access Memory (RAM) and analog interfaces are turned off. Some logic modules can remain active, providing limited functionality during sleep. These remaining units should be clocked from an external, extremely low-power 32.768 kHz clock (commonly found in watches). A 32.768 kHz crystal is preferred, as it is low-cost, commonly available and easy to use. The frequency of 32.768 Hz is commonly used because it is equal to $2^{15}$. By using a 15 stage binary counter, a precise 1 s period can be achieved. To further minimize $P_{\mathrm{static}}$ in transistors, the MCU operating voltage can be reduced. Some controllers include an on-chip voltage regulator, designed to lower the MCU voltage during sleep to 1.2 V.

Leakage on the IO pins of the controller, can be reduced by configuring unused pins to drive to a high or low state. Digital pins consume the least amount of power when the input voltage is near one of the used voltage rails. If the input voltage on digital ports is near the midpoint between $U_{SS}$ or $U_{DD}$, the transistors inside the IO port are biased in the linear region, consuming a significant amount of energy. The linear region of a transistor lies between cutoff and saturation. When a transistor is biased in the linear region, it effectively acts as a voltage controlled resistor. Used IO pins are best configured as high impedance inputs when going to a low power sleep state [85]. These configurations need to be set manually by the developer.

As a consequence of “sleeping as much as possible”, the MCU will need to preserve RAM and register values so it can continue after a sleep period. A microcontroller has special low-power RAM retention schemes to minimize leakage. This is typically achieved either by the use of a very low current latch biasing scheme or by the use of special retention latches that can hold the state without significant leakage [84].

IoT nodes are commonly equipped with replaceable batteries and/or unstable energy harvesting methods. As these can not provide a stable power source, Brown-Out Detection (BOD) will need to be enabled to monitor the supply voltage. To prevent the controller from getting into a corrupt state, the BOD will reset the controller as soon as the supply voltage drops below a certain minimum retention value. While this functionality consumes a small amount of power, it can be used to react on battery fluctuations. The node could, when low battery fluctuations are detected, stop consuming large amounts of energy by postponing packet transmission.

Most vendors offer different standby or sleep options: variations on RAM retention and BOD state. Modern low-power controllers also offer a varying amount of low-power integrated functions that remain active in certain sleep states, e.g., a RTC and low-power serial communication. Enabling these functions will increase the power consumption during sleep. Some examples are included in Table 4. It is obvious that unused and unnecessary device functions, are best left disabled to minimize power consumption.

#### 4.1.2. Power Management

Power provisioning and controlling is done by the controller and the on-board PMU (Figure 11). The design needs to incorporate mechanisms to switch off peripherals in order not to waste idle energy. This is certainly the case when entering sleep mode.

##### Microcontroller-Based Power Management

Low-current peripherals (<20 mA) can be directly powered from IO pins. The microcontroller can then completely power off the peripheral during sleep, thereby entirely eliminating any sensor sleep currents. For high-power hardware, such as radio modules, IO ports will not be able to support the current drawn in active mode. Therefore, external digital load switches can be incorporated in the design of the IoT node. The TPS22860 is an example of a “ultra-low leakage load switch” that can be used in IoT context. This specific load switch supports a continuous output current of 200 mA and has a leakage current of 100 nA [89]. The leakage current of these switches are important to minimize the energy drain in sleep mode. Another approach, when using multiple voltage rails for multiple peripherals, is to disable any unused voltage rail. By using an LDO with an enable pin, one could instantly switch off any peripherals, connected to that voltage rail. The LP2985 is such an device, only drawing 0.8 μA at most when in off mode [90]. All microcontroller-based power management has to be implemented by the developer through a careful hardware and firmware design.

##### PMU-Based Power Management

In contrast to the microcontroller-based mechanism, a PMU is built-in so no further hardware design is required. The PMU manages the power inside the controller, providing a constant supply voltage to components inside the controller. Additionally, the PMU controls the power distribution inside the controller, i.e., cut-off or provide power to certain blocks. By doing this, different power states are supported each providing different features. For the well-known Arm-based types of microcontrollers, that are much-used in IoT designs, these are the so-called energy modes.

Table 5 provides an overview of the energy modes provided by Silabs’ EFM32 Arm-based line of micrcontrollers. The goal of these energy modes is to offer an easy way for programmers to trade between energy-efficiency and functionality. A “lower” energy mode will offer more functionality, but at the cost of more energy being spent.

- When the CPU is executing code, the controller is in energy mode 0. All peripherals can be enabled and the clock speed can be throttled to speed up processing. Enabling extra peripherals will, of course, further increase the current draw,
- In energy mode 1, the CPU clock is disabled. However, all peripherals are still available and they can be configured to work without intervention of the CPU. For example, a timer can be used to periodically start sampling of the ADC which, in turn, uses Direct Memory Access (DMA) to store its samples immediately into memory for later processing by the CPU,
- This mechanism can not be used in energy mode 2, as no high-frequency clocks are available. Only low-power peripherals are available and periodic wake-ups of the CPU can be triggered by using a RTC, running on a low-frequency oscillator,
- In energy mode 3, this RTC is disabled. This means that the CPU can only be woken from an external source, e.g., a pin interrupt on the GPIO. However, data is still being retained in RAM.
- Energy mode 4 is the highest energy efficiency mode, offering almost no functionality. The system can only be woken up by GPIO interrupt.

Whenever an interrupt occurs, e.g., a timer fires, the controller will be setup back to EM0. This allows the CPU to process the interrupt, and eventually, go back to a higher energy mode. To ensure this way of working is energy-efficient and practical, switching between energy modes takes only a few microseconds.

#### 4.1.3. Sensors

When the use case only demands periodic sensor measurements, a sensor can be put in sleep mode for the entire duration of the sleep period. Interrupt-based sensors should be employed to enlarge the sleep period, and hence, no or limited busy waiting needs to be performed. More details regarding the wake-up capabilities of sensors is discussed in Section 4.2.2. Typically sensors can be put in sleep-mode through driving an IO pin or through a digital interface, e.g., SPI or I2C. In the latter case, the analog circuitry and other blocks are powered-down, while the digital interface remains powered to still receive commands from the controller during sleep. Based on the application requirements, the sampling frequency should be minimized to extend the sleep period of the sensing device. This again is a responsibility of the developer.

#### 4.1.4. Radio Module

Depending on the IoT connectivity technology, radio modules include low-power or sleep states between transmissions. Besides this, several measures can be taken to further minimize the power consumption of the radio module during sleep. It is important to select radio modules with a low-power consumption in all states, but especially in the sleep state, as presented in [92].

When using LoRaWAN or Sigfox, modems can generally be turned off completely (e.g., by using a load switch in series with the radio module). When using OTAA in LoRaWAN, it is important to store the parameters obtained during the join mechanism in non-volatile memory. In this manner, a potential join request each time the IoT nodes wakes, is eliminated. As illustrated in [1], nodes can go out of spec by forcing the node to enter sleep mode prior to opening the receive windows. Note that this approach deviates from the intended protocol specification, yet can yield significant energy reduction.

When using NB-IoT, the radio module should be put in Power Saving Mode as long and as frequently as possible, as this is the deepest sleep mode possible. In PSM, the connection parameters are maintained so the previous connection can be quickly re-established. These connection parameters are network dependent and cannot be saved in non-volatile memory, as periodic updates are required (Tracking Area Update (TAU) updates). It is therefore not advisable to completely turn off the NB-IoT modem, as any interruption would result in having to reconnect to the network (thus wasting energy). Notably, this is application-dependent and it is up to the developer to determine the most optimal approach.

### 4.2. Wake-Up

After a sleep period, the controller wakes-up to start a measurement and optionally transmits a message. During the wake-up, sensors warm-up, crystals have to stabilize, and radio modules will optionally reconnect to network. The trigger, instantiating the wake-up, could originate from a clock source, external event, comparator event or even from an RF signal through the use wake-up radios [10,75]. Depending on the previous state, i.e., sleep or power-down, the wake-up time and resulting energy consumption is different.

#### 4.2.1. Controller

When waking from sleep, the microcontroller effectively re-enables its high-speed system clock. When choosing a clock source, it is better to choose an accurate, quick-starting, on-chip oscillator instead of an external crystal oscillator. The IoT node should support waking-up from sleep mode from either an external trigger (sensor interrupt) or an internal timer. The most flexible periodic wake-up source is an integrated RTC. Depending on the time-critical nature of the application, the RTC can be run from either an external crystal oscillator (for extra accurate timing) or from a low-cost internal oscillator. This RTC enables the core of the microcontroller to be put to sleep for a certain period of time, after which it continues the operation. By using an external trigger source, the controller can go into a deeper energy saving state by also deactivating RTC timers. This lowers the energy expenditure of the microcontroller but requires that the external sources are powered. A common example is the use of an accelerometer, where an interrupt is fired on an input pin of the controller when a certain event is detected. As stated, this allows the controller to enter a deep sleep state, but the sensor still needs to remains active.

#### 4.2.2. Sensors

**Figure 15:**
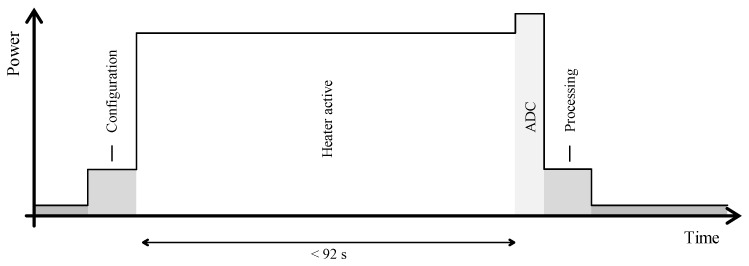
Power graph depicting the workings of a ’forced’ gas measurement with a BME680 sensor [47]. A sensor remains in sleep and has no active operations until the controller commands, or forces, a measurement. A heated bed is required for an accurate gas value measurement. Heating the sensor element can take up to 92 s, consuming large amounts of energy.

When waking sensors from sleep, it is important to allow enough time for analog circuitry to stabilize prior to measuring. Some electronic components like voltage regulators or voltage references can have a major impact on the settling time. They require significant external decoupling capacitors, and can take milliseconds to settle. Most often, vendors will only quote wake-up times for the digital circuitry, while ignoring the time it takes for the analog circuitry to settle. Some sensors inherently need a longer time to wake, e.g., volatile gas sensors and GPS sensors. Volatile gas sensors (like the Bosch BME680) require the sensing element to be heated up to 320 ${}^{\circ }C$ before being able to correctly perform measurements. These heaters can draw a significant amount of current, up to 13 mA at 1.8 V [47]. Heating the sensing element to the appropriate temperature can take up to 92 s. The combination of larger heater currents and slow response times results in a high energy consumption per gas measurement (Figure 15).

GNSS receivers are also sensors that can potentially require large amounts of energy. They typically can take over 30 s to perform a ’cold’ accurate position measurement [93], while drawing considerable currents. A cold GPS measurement starts without any additional location information: no satellite information and no course or previous location. The U-Blox ZOE-M8B (marketed as super low power), draws 34.5 mA (at 1.8 V) while obtaining an accurate GPS fix. To illustrate, one accurate GPS location measurement requires 1.86 J, while one LoRaWAN transmission, in the worst-case setting (SF12/51bytes), consumes only 1.17 J.

Several techniques, however, exist to reduce the energy consumption of a GPS sensor. Assisted Global Positioning System (A-GPS) attempts to shorten the satellite acquisition time reducing the wake-up time. A course satellite position is requested in the cloud and loaded into the sensor, thereby reducing the satellite search space and reducing the Time To First Fix (TTFF) [48]. Despite these energy improvements, some applications do not require a very accurate localization. An estimate of the area or region in which a sensor is deployed, is sometimes acceptable. Some LPWAN technologies feature such a coarse-grained localization service. These techniques employ the received data packets to estimate the location of the node. No further signaling is required. Sigfox and LoRaWAN are able to support localization services [94]. For example, Sallouha et al. [95] demonstrate how the LPWAN communication itself can serve as a means of determining whether or not nodes are deployed in each others vicinity. These native geolocalization techniques do not require any wake-up time as was present with GNSS systems.

### 4.3. Active

During the active state the required peripherals and controller are powered-up and active to start or continue the operation. In this phase, one or more sensors are measured and processed. As illustrated by Figure 3, in conventional connected sensor systems energy expenditure of processing is much lower than transmitting a packet. As a result, we propose the approach of “think before you talk” (Figure 14a). It involves on-node processing to verify the meaningfulness of the measured data prior to transmission. It also means that any compressing is advised in order to minimize the payload size of the uplink packet. Accumulating non-time critical sensor measurements could even further lower the energy impact of the transmit phase.

#### 4.3.1. Controller

Optimizing energy consumption in the MCU of an IoT device, is often directly related with the “race to sleep” strategy. This can be achieved by using a high clock frequency and dedicated hardware to perform operations more efficiently. A controller can then sooner enter the sleep state, reducing the amount of time in the active state. The power consumed by the controller is directly proportional to the switching frequency, as shown in Equation (3). Manufacturers therefore normalize the quoted current draw to a current per frequency (commonly μA/Hz) basis for a specific supply voltage. Most often, the frequency specified represents the system clock frequency. The real performance metric however is the instruction speed: the amount of system clock pulses it takes for certain low level operations to complete. The system clock can run at twice (or more) the speed of the actual instructions, doubling (or more) the effective power consumption.

Most microcontrollers integrate an internal clock divider and/or Phased Locked Loop (PLL). This lets the software engineer, respectively, slow down or speed-up the system clock. When no high speed operation is needed, a lower system clock can be selected. When high computational power is required, an integrated PLL could be used to boost the clock speed, thereby reducing the time needed for an instruction.

In some controllers, several standard operations can be offloaded to additional integrated hardware components. Security and Cycle Redundancy Check (CRC) calculations are prime examples. By implementing these features in hardware, a lot of computational burden is offloaded from the CPU to a dedicated hardware block. This effectively reduces the amount of CPU cycles it takes to perform these calculations. The CPU will need to wait for the calculation to be completed in hardware. This time is dependent on the calculation itself, but is shorter than any implementation using the CPU [96].

While waiting for results of any peripheral, it is of high importance that the controller can go to a lower energy state, i.e., “sleep as much as possible”. Keeping the CPU active while waiting for results needs to be avoided. The response time of most sensors is typically lower than the instruction speed of a controller. It is clear that the longer the main CPU can spend in sleep, the more energy savings can be obtained. Three energy effective strategies can be implemented for retrieving the sensor data: polling data transfer, interrupt driven data transfer and DMA [96]:

- Polling data transfer. Data is read using processor instructions. The controller actively polls the sensor for available date. When no data is available, the controller will wait for a pre-programmed amount of time. This time is better spent in a low-power sleep state. After this time, the controller will check again whether (sensor) data is ready. When data is available, it is transferred to memory or directly processed by the CPU,
- Interrupt driven data transfer. Data is read using processor instructions, but the controller does not need to check whether data is ready. When data is available, the sensor will interrupt any controller state by means of a changing GPIO pin. The controller will wake from sleep or interrupt its current process and acknowledge by starting the data transfer using the CPU,
- Direct Memory Access. Data is read from the peripheral, not by using processor instructions but by using the integrated Direct Memory Access Controller (DMAC). This will transfer the incoming data directly to a programmed memory block. The processor continues to wait in a low power until the DMAC calls an interrupt after the last byte is transferred. As IO operations are much slower than CPU operations, this results in a considerable energy saving.

#### 4.3.2. Radio Module

Figure 3 illustrates the large energy cost of transmitting data through a wireless interface. Processing power, however, does not require large energy contributions. Therefore, verifying the validity of the collected data can be crucial when operating on a limited energy budget. In a track and trace context, one could only transmit locations when motion is detected on the IoT node. Furthermore, the accuracy of the location can be verified on-node by means of parameters such as the delusion-of-precision prior to transmitting an inaccurate position.

Sending larger messages through an LPWAN communication link generally requires more time to be transmitted, thus spending more energy sending one message. The application payload size of one cycle should be kept at the minimum. It is inadvisable to add extra unnecessary static overhead bytes. When designing for one specific application, a byte numbered protocol could be put in place. An example is the Cayenne Low Power Payload format. Sensor types are allocated to predefined hex symbols in order to minimize the number of bytes. Despite the convenience of such a payload format, defining your own dedicated format could achieve even lower payload sizes. Depending on the required range and accuracy of the sensor measurement, the number of bits per sensor reading can be reduced.

Energy consumption of IoT communication, rises with higher payloads. However, due to protocol overheads, the energy cost per byte lowers when sending large payloads. Depending on the required response time, several sensor measurement intervals can be accumulated into a single message. This approach lowers the average energy cost per sensor measurement, as presented in [18].

#### 4.3.3. Power Manager

**Figure 16:**
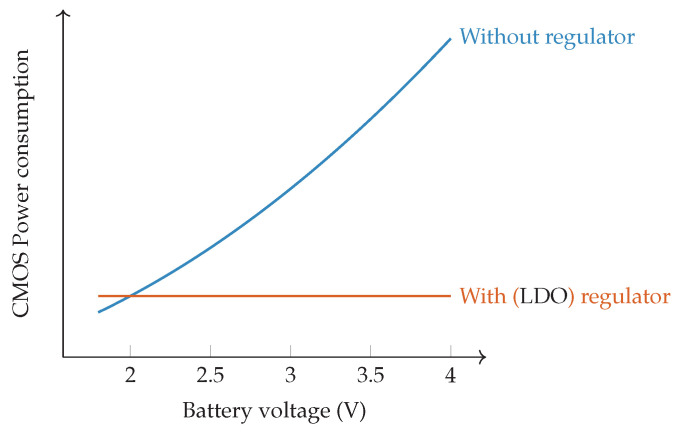
Illustration of possible power savings by using a fixed voltage, provided by an LDO voltage regulator [84].

As demonstrated in Equation (3), power consumption in CMOS circuitry can be divided in $P_{\mathrm{switch}}$ and $P_{\mathrm{static}}$. When the controller is active, the power consumption will be predominantly determined by $P_{\mathrm{switch}}$. This is the switching or active power and refers to the power consumption due to the switching of the digital logic. The supply voltage $U_{\mathrm{DD}}$ has a quadratic relation to the overall power consumed. When dealing with voltage sources significantly higher than the operating voltage, adding voltage regulation can yield significant power savings. The voltage regulation will provide a lower, steady supply voltage to the IoT node. By using a voltage regulator with a low quiescent current (e.g., Texas Instruments LP2985), energy savings can be obtained at almost all battery voltages, as depicted in Figure 16.

Switching type converters may be a possible solution, best suited for applications requiring a large voltage conversion ratio from the input voltage $U_{\mathrm{in}}$ to the required voltage supply $U_{\mathrm{DD}}$. These converters offer a higher energy efficiency than a typical LDO linear voltage regulator. However, the sleep current of a switching type converter will be significantly higher than an LDO due to the constant switching of the converter. A lower complexity, low-cost and low-power LDO voltage regulator can be the better choice for a battery operated IoT node. The average voltage conversion is small (ideally approaching 1:1 at the end of the battery life) [84].

### 4.4. Exemplary Use Case: Acoustic Event Detection

Application context Consider the case where, in an urban setting, acoustic noise pollution needs to be detected. The application needs to detect whether noise levels are above a certain threshold and, if possible, provide information about the nature of the sound, e.g., music, explosion, mass panic, etc. To that end, wireless IoT nodes equipped with a microphone are dispersed in the city. To eliminate the need for cables, they are battery-powered (possibly assisted by energy harvesting through solar power). In any case, energy consumption needs to be carefully managed such that sufficient remains available at all times in order to have reliable detection whenever an acoustic event occurs.

#### 4.4.1. How to Sense?

In this particular application, a microphone with (adjustable) wake-on-sound functionality can lead to significant energy savings, even if it will in this wake-on-sound mode consume considerably more energy compared to one that is completely turned off. To explain this trade-off, we inspect how an IoT node equipped with a regular low-power microphone would handle the detection of acoustic events. The microcontroller would have to periodically wake up the microphone (and amplifiers) to record audio and process it, i.e., determine whether the recorded sound level is above a set threshold. To make sure that even short events are detected reliably, this process would have to take place, say, every second. Even with the microcontroller’s RTC running, which is needed for the periodic wake-ups, the system consumes an order of magnitude less power in sleep, compared to a system that uses a microphone which is continuously in wake-on-sound mode (see Table 6). In this particular example we consider the Vesper VM1010, which can be configured to wake up when the sound level is over a certain threshold. The threshold can be set fixed or even adjusted on the fly [97]. The wake-on-sound operation mode avoids the need to record and process all sound sequences, which requires several orders of magnitude more power. The latter is required in the operation mode where a completely shut off microphone is periodically woken up. Hence, it is still more energy-efficient to use a microphone in wake-on-sound mode in situations where acoustic events that sporadically exceed a certain threshold and may be short, need to be detected.

#### 4.4.2. Choosing a Wireless Technology

As the application is event-driven and events originate from the device, an uplink-focused technology is best selected. In case on-node classification of sound sources is infeasible, a higher throughput technology as NB-IoT is required to transmit the full recording. However, if the uplink data only contains events or the classification can be done locally, both Sigfox and LoRaWAN can suffice. If the nodes are located on the same site, a private LoRaWAN network can be set-up. However, in case the nodes are spread over different locations, an LPWAN operator provides connectivity. For example, consider a 12 B packet—holding information about the acoustic event—being sent at different intervals. The energy per packet and the duration of the transmit mode for the considered LPWAN technologies is depicted in Table 7. Due to the long transmit time of Sigfox and the long initialization time of NB-IoT, these technologies are less energy-efficient than LoRaWAN—in particular for low-periodic events. Moreover, the intuitive approach of shutting-down the NB-IoT model between transmission is not beneficial compared to utilizing the Power Saving Mode to mitigate reconnecting to the network.

## 5. Conclusions and Outlook

Low-Power Wide-Area Network technologies are optimized for low-power and remote connections. Networks are now deployed operating both in unlicensed spectrum and in cellular licensed bands. Still, a meticulous design and disciplined operation of the IoT nodes is essential to provide them a long autonomy. Furthermore, in order to allow the deployment of IoT nodes on an increasingly larger scale, extending the battery lifetime of these node is imperative. Due to the high number of devices, the maintenance and environmental impact [100,101] becomes in-negligible.

In this work, we elaborated on the manners in which to support long-range wide-area connections and how they are implemented in LPWAN technologies. These PHY and MAC schemes need to be accommodated with hardware and software tailored to the intended applications. Based on the characterization of the anatomy of an IoT node, the required hardware considerations to achieve a low-power operation are discussed. Energy-efficient microcontrollers, energy harvesting techniques, energy storage technologies and others are reviewed based on their energy expenditure or contribution. Besides a careful IoT node design, strategies to extend the operation time of the node is discussed. This primarily involves applying mechanisms in the firmware and requires a detailed knowledge of the underlying layers and components. We conclude with an illustrative example of a real-world application on how the proposed design principles and choices could increase the autonomy of the IoT node.

Researchers are investigating to upgrade LPWAN technologies. In particular, the deployment of massive MIMO technology, that leverages on large antenna arrays, is very promising to save transmit energy for remote IoT nodes [102]. This can considerably lower a major obstacle to prolong the battery life of remote IoT nodes, as explained in this paper. Furthermore, researchers propose different mechanisms to optimize conventional LPWAN technologies [18,41,103]. Multiple Radio Access Technology (Multi-RAT), adopted from cellular standards, is introduced to intelligently switch between LPWAN technologies in order to dynamically adapt to the current application requirements and operating environments [104,105]. Besides an adaptive multi-radio approach, low-energy nodes will have to become self-diagnostic, self-recovering [106] and have a low environmental impact [107]. If no or only limited energy harvesting is possible and IoT nodes cannot be equipped with large enough batteries, energy can be provisioned through Unmanned Vehicles [108]. Furthermore, these sensors may get embedded in difficult to reach places, making it cumbersome for human intervention. By using these UVs, sensors equipped with small batteries at remote locations can be conveniently recharged, enlarging their operation time. Moreover, UVs can be used to collect data to further minimize the energy consumption and extend the area deployment [109]. Hence, we expect more UVs to be deployed in the context of IoT.

## Figures and Tables

**Table 1:** Overview of approaches of LPWAN technologies to address long-range and low-power requirements. The PHY and MAC is simplified in order to transmit small packets at a low data rate and low transmit power.

		LoRaWAN	Sigfox	NB-IoT
**PHY**				
	Modulation scheme	CSS	D-BPSK	BPSK/QPSK (SC-FDMA)
	Frequency	868 MHz	868 MHz	GSM (e.g., 900MHz) LTE (e.g., 1700MHz)
	Bandwidth	250 kHz and 125kHz	100 Hz	200 kHz
	Transmit Power (dBm)	ISM governed: max. 14 (node) /27 (gateway)		14/20/23
**MAC**				
	Protocol overhead			
	Initial Access	None (ABP)/Low (OTA)	None	High
	Uplink Packet	13–28 bytes	14 bytes	IP-based (depends on higher layer protocols)
	Collisions			
	Freq. div.	No	Yes	
	Space div.	Yes	Yes	n.a. (grant-based)
	Time div.	No	Yes	
	Overhearing	No	No	No (grant-based)
	Adaptive PHY control	Yes (ADR)	No	Yes (CE levels)
	Maximum payload size	∼250 bytes	12 bytes	1600 bytes

**Table 2:** Examples of IoT development platforms.

	MKR WAN 1300	The Things Uno	Seeeduino LoRaWAN	ST B-L072Z-LRWAN1
Host MCU	SAMD21	ATmega32u4	ATSAMD21	STM32L0
MCU Architecture	Arm M0+	AVR 8 bit	Arm M0+	Arm M0+
MCU Clock speed	48 MHz	16 MHz	48 MHz	32 MHz
Modem	Murata CMWX1	Microchip RN2483	RisingHF RHF76	Semtech SX1276
Operating voltage	3.3V	5V	5V	3.3V
Power usage	Low	High	Medium	Low
Price indication	€33	€55	€47	€42

Note that the ST B-L072Z-LRWAN1 features the Murata CMWX1ZZABZ-091 module, integrating both microcontroller, modem and RF front-end on one module.

**Table 3:** Overview of the most commonly used energy storage technologies for IoT applications. The discharge rate is described by the C rate. A battery with a C rate of 1 and a capacity of 1 Ah can supply 1 A for 1 h.

		Non Rechargeable					Rechargeable	
Composition		Alkaline	Lithium Thionyl Chloride	Lithium Manganese Dioxide	Lithium Iron Disulfide	Lithium Poly Carbon	Lithium Cobalt Oxide	Lithium Polymer
Abreviation		ALK [50]	LTC [51]	LMD [52]	LID [53]	PC [54]	LCO [55]	LIPO [56]
Volumetric energy density	Wh/L	506	1080	683	562	532	602	309
Weight energy density	Wh/kg	176	480	323	300	300	217	185
Power density	W/kg	18	<0.1	6.5	300	0.3	650.2	123
Internal resistance	mΩ	<250	High	10–70	<350	<1000	∼40	∼40
Nominal voltage	V	1.5	3.6	3	1.5	3	3.65	3.7
Discharge cut-off voltage	V	0.9	2	1.5	0.9	1.5	2.5	2.75
Discharge rate	-	C/10	C/10,000	1 C	1 C	C/1000	0.5–1 C	1 C
Self discharge	%/year	2–3	<1	1	2	0.5	12	60
Operating temperature	${}^{\circ }C$	−10 to 50	−55 to 85	−40 to 60	−40 to 60	−40 to 85	−20 to 60	−10 to 50
Price	EUR/Wh	∼0.1	∼1	∼0.7	∼0.5	∼1	∼0.3	∼1.8
Shelf life	year	7–10	10	10–20	10	10	∼10	<5

**Table 4:** Overview of typical current drawn by optional peripherals in sleep mode. MCU supply voltage is 3.3 V.

Parameter	Arm M0+ (ATSAMD21) [86]	8-bit PIC (PIC16F1717) [87]	16-bit PIC (PIC24FJ128GA310) [88]
BOD (μA)	0.132	0.8	0.07
WDT (μA)	0.007	0.5	0.8
32 kHz RTC (μA)	0.056	1.3	0.4

**Table 5:** Energy modes provided by Silabs’ EFM32 Arm-based line of microcontrollers [91]. The listed current consumption is a minimum and increases further with each enabled peripheral. Because high frequency clocks are enabled (and required) in EM0 and EM1, the current draw is a function of the clock frequency.

Energy Mode	Current Consumption	Capabilities (Non-Exhaustive List)
**Energy Mode 0**	180 μA/MHz	Full capabilities.
Active/Run Mode		High performance CPU and with all peripherals available (if enabled)
**Energy Mode 1**	45 μA/MHz	CPU disabled, all peripherals available (if enabled)
Sleep Mode		PRS combined with DMA enables data transmission between peripherals
		without CPU intervention
**Energy Mode 2**	0.9 μA	No high frequency oscillators, which means not timers or continuous
Deep Sleep Mode		sampling of the ADC. RTC on a low-frequency oscillator.
		Low-Energy UART, I2C (slave operation), Analog Comparator, GPIO
**Energy Mode 3**	0.6 μA	Full RAM retention, no RTC
Stop Mode		Watchdog timer, I2C slave operation, GPIO, Analog Comparator
**Energy Mode 4**	20 nA	No RAM retention, only asynchronous wake-ups possible from
Shutoff Mode		GPIO or reset.

**Table 6:** Comparison of sensing mechanisms for the acoustic event detection use case discussed in Section 4.4. Power estimates based on EFM32 controller and VM1010 microphone.

**a**. Power consumption in each mode.		
**Mode**	**CPU State**	**Power**
sensor off	EM2	5 μW
wake-on sound	EM3	50 μW
sample	EM1 @ 1 MHz + ADC	1 mW
process	EM0 @ 24 MHz	10 mW
**b**. Average power in μW (sampling and processing each take 10 ms).		
**Events/Hour**	**with Wake-on-Sound**	**Periodic Wake-Up**
3600	159	115
1200	86	115
100	53	115
10	50	115

**Table 7:** Power and energy consumption of Sigfox, LoRaWAN and NB-IoT for 12 B payload. The node is put to EM2 during transmission.

	LoRaWAN [39,98] (SF7)	LoRaWAN [39,98] (SF12)	Sigfox [37]	NB-IoT [99] (PSM)	NB-IoT [99] (shutdown)
Energy per packet	10.8 mJ	224.93 mJ	25.9 mJ	63.48 mJ	3275.16 mJ
Duration of packet	2.088 s	3.51 s	6.24 s	0.37 s	23.72 s
Transmission Frequency	Average power (μW) during one cycle				
1/15 min	17.0	254.9	28,797.7	70,908.2	3,639,071.5
1/hour	8.0	67.5	7203.1	18,008.3	909,771.6
2/day	5.2	10.2	604.8	1844.4	75,818.89
1/day	5.1	7.6	304.9	1109.7	37,911.9
0.5 /day	5.0	6.3	155.0	742.4	18,958.5

## Abbreviations

The following abbreviations are used in this manuscript:

A-GPS	Assisted Global Positioning System
ABP	Authentication By Personalisation
ADC	Analog-to-Digital Converter
ADR	Adaptive Data Rate
AES	Advanced Encryption Standard
ALK	Alkaline
AT	ATtention
AWGN	Additive White Gaussian Noise
BOD	Brown-Out Detection
BPSK	Binary Phase Shift Keying
CE	Coverage Enhancement
CMOS	Complementary Metal-Oxide-Semiconductor
CPU	Central Processing Unit
CRC	Cycle Redundancy Check
CSS	Chirp Spread Spectrum
DBPSK	Differential Binary Phase Shift Keying
DMA	Direct Memory Access
DMAC	Direct Memory Access Controller
ECC	Elliptic-Curve Cryptography
EDLC	Electric Double-Layer Capacitor
eDRX	Extended Discontinuous Reception Mode
EM	Electromagnetic
FFT	Fast Fourrier Transfomation
FSK	Frequency-Shift Keying
GNSS	Global Navigation Satellite System
GPIO	General-Purpose Input/Output
GPS	Global Positioning System
GSM	Global Systemfor Mobile Communications
I2C	Inter-Integrated Circuit
IMU	Inertial Measurement Unit
IO	Input/Output
IoT	Internet of Things
ISM	Industrial, Scientific and Medical
LCO	Lithium Cobalt Oxide
LDO	Low-Dropout Regulator
LED	Light Emitting Diode
LIC	Lithium-ion Capacitor
LID	Lithium Iron Disulfide
LIPO	Lithium Polymer
LMD	Lithium Manganese Dioxide
LoRa	Long Range
LoRaWAN	Long-Range Wide-Area Network
LPWAN	Low-Power Wide-Area Network
LPWANs	Low-Power Wide-Area Networks
LTC	Lithium Thionyl Chloride
LTE	Long Term Evolution
LTO	Lithium Titanium Oxide
M2M	Machine-to-Machine
MAC	Medium Access Control
MCL	Maximum Coupling Loss
MCU	Microcontroller Unit
MPPT	Maximum Power Point Tracking
MTC	Machine-Type Communication
Multi-RAT	Multiple Radio Access Technology
NB-IoT	Narrowband IoT
OFDMA	Orthogonal Frequency Division Multiple Access
OTAA	Over The Air Authentication
PA	Power Amplifier
PAPR	Peak-to-Average Power Ratio
PC	Lithium Poly Carbon
PCB	Printed Circuit Board
PHY	Physical
PLL	Phased Locked Loop
PMU	Power Management Unit
PRS	Peripheral Reflex System
PSM	Power Saving Mode
PV	Photovoltaic
QAM	Quadrature Amplitude Modulation
QPSK	Quadrature Phase Shift Keying
RAM	Random-Access Memory
RF	Radio Frequency
RTC	Real Time Clock
SC-FDMA	Single-Carrier Frequency Division Multiple Access
SHA	Secure Hash Algorithm
SNR	Signal-to-Noise Ratio
SOC	System on Chip
SPI	Serial Peripheral Interface
TAU	Tracking Area Update
TPMS	Tire-Pressure Monitoring System
TTFF	Time To First Fix
UART	Universal Asynchronous Receiver/Transmitter
UV	Unmanned Vehicle
VOC	Voltatile Organic Compound
